# Nutrient adequacy of Japanese schoolchildren on days with and without a school lunch by household income

**DOI:** 10.29219/fnr.v64.5377

**Published:** 2020-12-11

**Authors:** Chika Horikawa, Nobuko Murayama, Hiromi Ishida, Taeko Yamamoto, Sayaka Hazano, Akemi Nakanishi, Yumi Arai, Miho Nozue, Yukiko Yoshioka, Saori Saito, Aya Abe

**Affiliations:** 1 Department of Health and Nutrition, University of Niigata Prefecture, Niigata, Japan; 2 Department of Applied Nutrition, Kagawa Nutrition University, Saitama, Japan; 3 Department of Nutrition, Kanagawa University of Human Services, Kanagawa, Japan; 4 Department of Health and Nutrition, Matsumoto University, Nagano, Japan; 5 Department of Health and Nutritional Sciences, Tokoha University, Shizuoka, Japan; 6 Department of Nutritional Management, Sagami Women’s University, Kanagawa, Japan; 7 Department of Health and Dietetics, Teikyo Heisei University, Tokyo, Japan; 8 Graduate School of Humanities, Tokyo Metropolitan University, Tokyo, Japan

**Keywords:** Japan, household income, schoolchildren, nutrient adequacy, dietary reference intakes

## Abstract

**Background:**

Evidence for whether the nutrient intakes of Japanese schoolchildren differ according to household income is sparse.

**Objective:**

We investigated the role of school lunches for nutrient adequacy among Japanese primary school children using dietary reference intakes in a cross-sectional survey.

**Design:**

Participants were 10- to 11-year-old (5th grade) children from 19 public primary schools in four prefectures of East Japan, and 836 children were analyzed. The participants completed 24-h dietary records with photographs of their meals for 4 consecutive days, composed of 2 days with and 2 days without a school lunch. −Children’s household income was obtained from questionnaires that were completed by the participants’ guardians and divided into the following three categories: low (0.2236–2.2361 million yen; *n* = 319), middle (2.3333–2.8868 million yen; *n* = 194), and high (3.1305–6.3640 million yen; *n* = 323). Logistic regression analyses were used to estimate the odds ratios for whether participants had poor nutrient intakes, with adjustment for confounders.

**Results:**

On days without a school lunch, the prevalence of nutrient shortages was significantly higher compared with those on days with a school lunch for most macro- and micronutrients among all three levels of household income. Children from low-income households had higher rates of nutrient shortages for vitamin B_6_, pantothenic acid, K, Mg, P, Fe, and Zn than those from middle-income households on days without a school lunch (*P* = 0.004, 0.001, 0.001, 0.006, 0.037, <0.001, and 0.015, respectively), but those differences were not significant on days with a school lunch.

**Conclusion:**

The findings suggest that school lunches are important for achieving adequate nutrient intakes in schoolchildren and reduce disparities of adequate nutrient intake by household income levels.

## Popular scientific summary

- We investigated the role of school lunches for nutrient adequacy among Japanese primary school children.
- On days without a school lunch, the prevalence of nutrient shortages was higher than those on days with a school lunch irrespective of income.
- Children from low-income households had higher rates of nutrient shortages than those from middle-income households on days without a school lunch.
- School lunches are important for achieving adequate nutrient intakes in schoolchildren and reduce disparities by income.

Socioeconomic disparities are a serious global concern, especially since children from low-socioeconomic status (SES) families are more likely to be obese (1). Childhood obesity has also been associated with adulthood obesity and biomarkers in adults that are associated with the metabolic syndrome, cardiovascular disease, and several cancers (2). Children undergo rapid biological and cognitive changes (3) in addition to forming dietary habits that persist into adulthood (4). It is essential to establish evidence that defines the desirable food and nutrient intakes for children. Children from low-SES households in Western countries have less healthy food and nutrient intakes than those from middle- or high-SES households, including higher intake of energy-dense foods such as meat products and sugar-sweetened drinks, and lower intake of fruits, vegetables, fish, and low-fat milk (5, 6). A systematic review revealed positive associations between a low SES and low intake of micronutrients such as vitamin B_12_, folate, vitamin C, vitamin D, Ca, Fe, I, and Zn in Western children (7). In Asia, a study from Korea showed that children from low-SES households consumed less energy from protein and more energy from carbohydrates compared with those from high-SES households (8). However, the evidence for the relationship between SES and the adequacy of nutritional intake among Japanese children is sparse.

Although Japan was a relatively egalitarian society for several decades, the household income gap in Japan has widened since the 1980s (9). Previous studies on Japanese adults have reported associations of a low household SES with poor dietary intake and high morbidity and mortality from the 1990s onward (10). The socioeconomic disparities of Japanese children have also become greater, and the Japan Comprehensive Survey of Living Conditions reported the relative poverty rate of children to be 13.9% in 2015 (11). There is a limited amount of evidence regarding the association between SES and dietary and nutritional intakes in Japanese children. One study reported that the dietary intakes of preschool children in one city were not associated with household income after adjustment for maternal education (12). Our previous study of 10- to 11-year-old Japanese schoolchildren in four prefectures showed that a lower household income was related to a lower mean intake of fish/shellfish, sugar, protein, and several micronutrients in addition to a higher intake of carbohydrates (13). However, no studies in Japan have investigated whether schoolchildren differ in their nutrient adequacy according to their household income by considering dietary reference intakes (DRIs) (14), although shortages of nutrient intakes could affect the physical and mental growth of children during school ages.

Additionally, our previous study reported that the associations between income and food/nutrient intake were not significant on days with a school lunch but were significant on days without a school lunch (11). During the fiscal year 2018, elementary schools in Japan provided school lunches to 99.1% of all schoolchildren and offered the lunch service on approximately 190 days per year (15). Daily school lunches improve children’s nutrition based on the Standards for the School Lunch Program, according to the School Lunch Program Act, which was enacted in 1954 (16, 17). This standard prescribes the minimum requirement for energy and nutrition in daily school lunches, which must contain at least a third of children’s energy and nutritional requirements per day, and stringent requirements are prescribed for vitamin A (≥40%), vitamin B_1_ (≥40%), vitamin B_2_ (≥40%), Ca (≥50%), Fe (≥40%), and fiber (≥40%) (17). Previous studies from the United States have shown that the introduction of a school lunch program modified food security and weight status in schoolchildren (18, 19). Therefore, it is necessary to consider the adequacy of nutrient intake of schoolchildren as according to household income by assessing it separately for days with school lunches and days without school lunches.

In the present study, we aimed to investigate the association between household income and nutrient adequacy among Japanese primary school children using DRIs, and determine whether their nutrient adequacy differed between days with and without a school lunch.

## Methods

### Study participants

Participants were all 10- to 11-year-old (5th grade) children from 19 public primary schools in four prefectures of East Japan. The schools were located in areas A (three schools in two cities), B (six schools in one city), C (eight schools in one city and one village), and D (two schools in one city). A and C were rural residential areas, while B and D were urban residential areas. These prefectures were the areas in which the researchers’ workplaces were present; two were in metropolitan East Japan and two were in non-metropolitan East Japan. The total number of elementary schools in these four prefectures of East Japan is 2,526. Researchers asked each prefectural and city board of education to introduce them to elementary schools that might possibly participate in this study, and 19 school agreed to participate. The schools were selected after obtaining permission from the respective Boards of Education of the cities and head teachers of the primary schools. The protocol for the study, which is in accordance with the Declaration of Helsinki and the Ethical Guidelines for Clinical/Epidemiological Studies of the Japanese Ministry of Health Labor and Welfare, received ethical approval from the ethics committee of the University of Niigata Prefecture, Japan (No. 1309). A written informed parental consent was obtained for all subjects. The survey was carried out from September to December 2013.

**Fig. 1:**
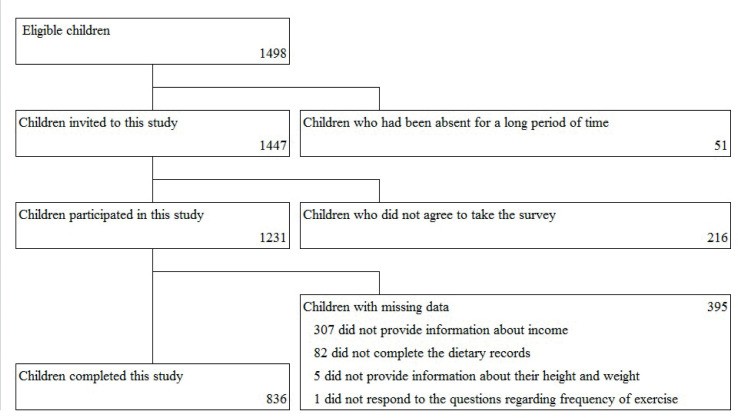
Flow diagram of study participants.

The eligibility criterion was that all children be in the 5th grade of elementary school. This study included 1,447 of the eligible 1,498 pairs of children and guardians after excluding children who had been absent for a long period, and 1,231 (85.1%) pairs agreed to participate. Finally, 836 pairs (57.8%) completed the surveys and their data were analyzed. The flow diagram of study participants is shown in Fig. 1.

### Household socioeconomic status

The household SES of the participating children was determined from a questionnaire completed by their guardians. The SES of the participants was assessed from children’s household income and the education levels of their fathers and mothers. The questions about household SES included the total annual household income, including salary, social benefits, family allowance, and rental income of all household members during the fiscal year 2012. Participants selected one of the following answers: <1 million yen, between 1 and 2 million yen, between 2 and 3 million yen, between 3 and 4 million yen, between 4 and 6 million yen, between 6 and 8 million yen, ≥8 million yen, or do not wish to answer. Each response was calculated as an equivalent household income by dividing the income by the square root of the number of household members (20), and the midpoints of each income category were used as representative incomes, i.e. 0.5, 1.5, 2.5, 3.5, 5, 7, and 9 million yen (21). We divided the income variables into the following three categories: low (0.2236–2.2361 million yen; *n* = 319), middle (2.3333–2.8868 million yen; *n* = 194), and high (3.1305–6.3640 million yen; *n* = 323). The median incomes of each group were 1.75, 2.5, and 3.5 million yen, respectively. The number of household members and the highest educational level of each parent were also determined.

### Dietary records of children

Nutritional and food intakes were assessed by 24-h dietary records with photographs for 4 consecutive days, composed of 2 days with a school lunch (weekdays) and 2 days without a school lunch (weekend). Dietary record sheets for 4 days, a camera, a mat with a graded scale to confirm the size of the tableware and amount of food, and instructions on keeping records were distributed to each child. Participants recorded the names and amounts of all foods and beverages consumed in a day, and each meal was photographed on the provided mat. Guardians were asked to support and confirm the records. A trained researcher explained to all children in each classroom how they should record food and photograph their meals using the same training manual. The details about this manual were described elsewhere (13).

We used a standardized software for population-based surveys and nutrition counseling in Japan (EIYO-KUN version 7.0, 2nd edition, developed at the site of the Shikoku University Nutrition Database), which are based on the Standard Tables of Food Composition in Japan (22), edited by the Japanese Ministry of Education, Culture, Sports, Science, and Technology to calculate nutrient, food, and energy intakes. Registered dietitians analyzing the dietary intake identified the names and amounts of food from the 4-day records and examined the meal photographs based on the manual.

### Confounding factors of children

Other measurements in addition to the dietary survey included sex, frequency of exercise, and food allergies and/or food restrictions. This information was obtained by providing questionnaires for the children to complete together in the classroom. Children were asked about their frequency of exercise per week, including outdoor play and excluding exercise class at school, and could choose among the following four categories: ≥5 days/week, 3–4 days/week, 1–2 days/week, or 0 day/week. The presence or absence of food allergies and food restrictions was checked with the inclusion of clinical treatment. The height and body weight of children were measured at school according to the published guidelines (23) in September 2013.

### Statistical analysis

Categorical variables were expressed as percentages of participants, while numerical variables were expressed as means and standard deviation (SD) or 95% confidence interval (CI). The *χ*^2^ test was used to compare categorical data among household incomes, and numerical variables were examined using one-way analysis of variance. DRIs for 10- to 11-year-old boys and girls (14) were used to assess whether each child met his/her nutrient intake requirements. Daily nutrient shortages were evaluated according to the estimated average requirement (EAR), while nutrient adequacy was evaluated according to the adequate intake (AI) and the dietary goal (DG) for preventing non-communicable diseases.

We estimated the distribution of the habitual intake of energy and nutrients during the 2 days with school lunches and the 2 days without school lunches by the best-power method, using the HabitDist software (National Institute of Public Health, Wako, Saitama, Japan) (24, 25). The adequacy of nutrient intake was calculated based on the estimated distribution of intakes as described earlier. Fisher’s test was performed to assess whether the proportion of participants with an insufficient nutrient intake differed between the days with and without a school lunch. When the variables used to weight the cases contained zero values, we added 0.5 to each cell as previously described (26).

To assess whether the nutrient adequacy of the participants during the 2 days with and 2 days without school lunches varied according to their household income, logistic regression analyses were used to estimate the adjusted odds ratios (ORs) and 95% CIs. The ‘middle’-income category was used as the reference for the ‘low’- or ‘high’-income categories to verify whether or not the low-income group had a different level of eating habits than the middle-income group. These analyses were adjusted for prefecture (A, B, C, and D), school (19 schools), sex (men and women), BMI (continuous), exercise frequency (>5 days/week, 3–4 days/week, 1–2 days/week, and 0 day/week), and food allergy status (yes and no) of the child, with parental education (less than high school, high school, college, university/graduate school, and don’t know) as a covariate. All *P*-values were two-sided, and the significance level was <0.05. All statistical analyses were carried out using SPSS Statistics Version 23 (IBM, Armonk, NY, USA).

## Results

Characteristics and household incomes of the 5th grade children (836 participants; 48.9% boys) are shown in Table 1. Significant differences were observed among the three income groups in terms of their residency/socioeconomic features such as prefecture, educational levels of the father and mother, household annual income, and number of family members (all *P* < 0.001). There were no significant differences in gender, frequency of exercise (excluding physical education class), presence or absence of food allergy, height, body weight, BMI, and degree of obesity among the three household income levels.

**Table 1:** Characteristics of participants according to household income level: 5th grade children (10–11 years old) from 19 schools in four prefectures of Japan

Variable	Category	Total (*n* = 836)		High (*n* = 323)		Middle (*n* = 194)		Low (*n* = 319)		*P*^†^
		*n*	%	*n*	%	*n*	%	*n*	%	
Prefecture of residency	A	188	22.5	73	22.6	44	22.7	71	22.3	<0.001
	B	221	26.4	86	26.6	73	37.6	62	19.4	
	C	283	33.9	88	27.2	44	22.7	151	47.3	
	D	144	17.2	76	23.5	33	17.0	35	11.0	
Gender	Boys	409	48.9	159	49.2	99	51.0	151	47.3	0.712
	Girls	427	51.1	164	50.8	95	49.0	168	52.7	
Exercise of child (except physical education class)	>5 days/week	296	35.4	111	34.4	77	39.7	108	33.9	0.167
	3–4 days/week	296	35.4	116	35.9	53	27.3	127	39.8	
	1–2 days/week	199	23.8	78	24.1	54	27.8	67	21.0	
	0 day/week	45	5.4	18	5.6	10	5.2	17	5.3	
Food allergy of child	Yes	71	8.5	30	9.3	14	7.2	27	8.5	0.715
	No	765	91.5	293	90.7	180	92.8	292	91.5	
Educational level of father	Don’t know	36	4.3	1	0.3	3	1.5	32	10.0	<0.001
	Less than high school	48	5.7	11	3.4	9	4.6	28	8.8	
	High school	298	35.6	97	30.0	70	36.1	131	41.1	
	Vocational/junior college	161	19.3	46	14.2	48	24.7	67	21.0	
	University/graduate school	293	35.0	168	52.0	64	33.0	61	19.1	
Educational level of mother	Don’t know	5	0.6	1	0.3	0	0.0	4	1.3	<0.001
	Less than high school	17	2.0	1	0.3	5	2.6	11	3.4	
	High school	308	36.8	94	29.1	82	42.3	132	41.4	
	Vocational/junior college	385	46.1	148	45.8	88	45.4	149	46.7	
	University/graduate school	121	14.5	79	24.5	19	9.8	23	7.2	
Household annual income (million yen)	<1	6	0.7	0	0.0	0	0.0	6	1.9	<0.001
	1 to <2	36	4.3	0	0.0	0	0.0	36	11.3	
	2 to <3	61	7.3	0	0.0	0	0.0	61	19.1	
	3 to <4	103	12.3	0	0.0	2	1.0	101	31.7	
	4 to <6	275	32.9	3	0.9	157	80.9	115	36.1	
	6 to <8	215	25.7	180	55.7	35	18.0	0	0.0	
	8 and over	140	16.7	140	43.3	0	0.0	0	0.0	
Number of family members	2	20	2.4	7	2.2	2	1.0	11	3.4	<0.001
	3	93	11.1	25	7.7	29	14.9	39	12.2	
	4	373	44.6	164	50.8	128	66.0	81	25.4	
	5	230	27.5	98	30.3	0	0.0	132	41.4	
	6	81	9.7	20	6.2	28	14.4	33	10.3	
	7	32	3.8	7	2.2	6	3.1	19	6.0	
	8	6	0.7	2	0.6	0	0.0	4	1.3	
	9	1	0.1	0	0.0	1	0.5	0	0.0	
										P for
Physique of child		Mean	SD	Mean	SD	Mean	SD	Mean	SD	trend^‡^
	Height (cm)	142.7	7.0	143.0	6.4	142.2	7.3	142.7	7.4	0.393
	Weight (kg)	35.8	7.5	36.0	7.5	36.1	8.2	35.4	7.2	0.534
	BMI (kg/m^2^)	17.4	2.7	17.5	2.7	17.7	2.8	17.3	2.5	0.240
	Degree of obesity (%)^§^	−1.9	14.0	−2.2	14.4	-0.2	14.4	-2.6	13.4	0.159

† *P*-value for χ^2^ test among income levels.

‡ *P*-value for one-way analysis of variance test among income levels.

§ Degree of obesity (%) = (body weight [kg]−standard body weight for height [kg])/(standard body weight for height [kg]) × 100.

Table 2 shows the data for nutrient adequacy from the 2-day periods with and without school lunches. On days without a school lunch, the prevalence of nutrient shortages was significantly higher compared with those on days with a school lunch for most macro- and micronutrients among all three household income levels. A significant prevalence of nutrition excess was seen only for salt intake regardless of income level. On days without a school lunch, the children tended to have a significantly lower prevalence of excess salt intake than on days with a school lunch.

**Table 2:** The prevalence of nutrient shortages/excesses by household income level and their differences between 2 days with and without a school lunch: 5th grade children (10–11 years old) from 19 schools in four prefectures of Japan

Nutrient				High (*n* = 323)						Middle (*n* = 194)						Low (*n* = 319)					
	Cutoff value per day		Ref.	Days with school lunch		Days without school lunch		∆%		Days with school lunch		Days without school lunch		∆%		Days with school lunch		Days without school lunch		∆%	
	Boys	Girls		type	*n*	%	*n*	%	%	*P*	*n*	%	*n*	%	%	*P*	*n*	%	*n*	%	%	*P*
Energy	<2,250 kcal	<2,100 kcal	EER	233	72.1	265	82.0	9.9	0.004	157	80.9	167	86.1	5.2	0.218	249	78.1	282	88.4	10.3	0.001
Protein	>20% E	>20% E	DG	0	0.0	0	0.0	0.0	1.000	0	0.0	0	0.0	0.0	1.000	0	0.0	0	0.0	0.0	1.000
	<13% E	<13% E	DG	0	0.0	30	9.3	9.3	<0.001	0	0.0	6	3.1	3.1	0.002	0	0.0	24	7.5	7.5	<0.001
	<40 g	<40 g	EAR	0	0.0	2	0.6	0.6	0.218	0	0.0	3	1.5	1.5	0.069	0	2.1	4	1.3	-0.8	0.021
Fat	>30% E	>30% E	DG	121	37.5	165	51.1	13.6	0.001	84	43.3	112	57.7	14.4	0.006	94	29.5	107	33.5	4.1	0.306
	<20% E	<20% E	DG	0	0.0	0	0.0	0.0	1.000	0	0.0	0	0.0	0.0	1.000	0	0.0	0	0.0	0.0	1.000
Carbohydrate	>60% E	>60% E	DG	28	8.7	162	50.0	41.3	1.000	10	5.2	9	4.6	-0.5	1.000	8	2.5	18	5.6	3.1	0.070
	<50% E	<50% E	DG	8	2.5	34	10.5	8.0	<0.001	3	1.5	4	2.1	0.5	1.000	2	0.6	0	0.0	-0.6	0.218
Fiber	<13 g	<13 g	AI	106	32.8	243	75.2	42.4	<0.001	77	39.7	140	72.2	32.5	<0.001	123	38.6	250	78.4	39.8	<0.001
Vitamin A	<450 µg RAE	<400 µg RAE	EAR	3	0.9	177	54.8	53.9	<0.001	4	2.1	101	52.1	50.0	<0.001	27	8.5	203	63.6	55.2	<0.001
Vitamin D	<4.5 µg	<4.5 µg	AI	2	0.6	141	43.7	43.0	<0.001	0	0.0	100	51.5	51.5	<0.001	53	16.6	188	58.9	42.3	<0.001
Vitamin E	<5.5 mg	<5.5 mg	AI	53	16.4	52	16.1	-0.3	1.000	32	16.5	54	27.8	11.3	0.010	51	16.0	113	35.4	19.4	<0.001
Vitamin K	<120 µg	<120 µg	AI	5	1.5	57	17.6	16.1	<0.001	0	0.0	39	20.1	20.1	<0.001	8	2.5	83	26.0	23.5	<0.001
Vitamin B1	<1.0 mg	<0.9 mg	EAR	6	1.9	133	41.2	39.3	<0.001	9	4.6	98	50.5	45.9	<0.001	6	1.9	149	46.7	44.8	<0.001
Vitamin B2	<1.1 mg	<1.1 mg	EAR	4	1.2	159	49.2	48.0	<0.001	8	4.1	100	51.5	47.4	<0.0001	21	6.6	191	59.9	53.3	<0.001
Niacin	<11 mg NE	<10 mg NE	EAR	0	0.0	0	0.0	0.0	1.000	0	0.0	0	0.0	0.0	1.000	0	0.0	0	0.0	0.0	1.000
Vitamin B6	<1.0 mg	<1.0 mg	EAR	7	2.2	96	29.7	27.6	<0.001	4	2.1	50	25.8	23.7	<0.001	8	2.5	140	43.9	41.4	<0.001
Vitamin B12	<1.5 µg	<1.5 µg	EAR	0	0.0	0	0.0	0.0	1.000	0	0.0	1	0.5	0.5	1.000	0	0.0	1	0.3	0.3	1.000
Folate	<150 µg	<150 µg	EAR	0	0.0	9	2.8	2.8	0.004	0	0.0	13	6.7	6.7	<0.001	0	0.0	34	10.7	10.7	<0.001
Pantothenic acid	<6 mg	<6 mg	AI	11	3.4	180	55.7	52.3	<0.001	12	6.2	104	53.6	47.4	<0.001	36	11.3	221	69.3	58.0	<0.001
Vitamin C	<60 mg	<60 mg	EAR	3	0.9	45	13.9	13.0	<0.001	0	0.0	46	23.7	23.7	<0.001	2	0.6	86	27.0	26.3	<0.001
Salt	>6.5 g	>7.0 g	DG	320	99.1	293	90.7	−8.4	<0.001	190	97.9	179	92.3	−5.7	0.016	318	99.7	289	90.6	-9.1	<0.001
K	<1,900 mg	<1,800 mg	AI	1	0.3	84	26.0	25.7	<0.001	1	0.5	58	29.9	29.4	<0.001	4	1.3	138	43.3	42.0	<0.001
	<2,200 mg	<2,000 mg	DG	11	3.4	158	48.9	45.5	<0.001	6	3.1	92	47.4	44.3	<0.001	23	7.2	202	63.3	56.1	<0.001
Ca	<600 mg	<600 mg	EAR	52	16.1	277	85.8	69.7	<0.001	52	26.8	165	85.1	58.2	<0.001	84	26.3	289	90.6	64.3	<0.001
Mg	<180 mg	<180 mg	EAR	3	0.9	75	23.2	22.3	<0.001	4	2.1	56	28.9	26.8	<0.001	9	2.8	128	40.1	37.3	<0.001
P	<1,100 mg	<1,000 mg	AI	40	12.4	190	58.8	46.4	<0.001	29	14.9	125	64.4	49.5	<0.001	66	20.7	234	73.4	52.7	<0.001
Fe	<7.0 mg	<7.0 mg	EAR	129	39.9	203	62.8	22.9	<0.001	95	49.0	119	61.3	12.4	0.019	166	52.0	245	76.8	24.8	<0.001
Zn	<6.0 mg	<6.0 mg	EAR	2	0.6	17	5.3	4.6	0.001	1	0.5	11	5.7	5.2	0.006	1	0.3	34	10.7	10.3	<0.001
Cu	<0.5 mg	<0.5 mg	EAR	0	0.0	0	0.0	0.0	1.000	0	0.0	0	0.0	0.0	1.000	1	0.3	1	0.3	0.0	1.000
Mn	<3.0 mg	<3.0 mg	AI	259	80.2	292	90.4	10.2	<0.001	174	89.7	185	95.4	5.7	0.052	282	88.4	303	95.0	6.6	0.004

AI, adequate intake; DG, tentative dietary goal for preventing non-communicable diseases; EAR, estimated average requirement; EER, estimated energy requirement; %E, percentage of energy; NE, niacin equivalent; RAE, retinol activity equivalents; Ref. type, reference type.

Table 3 shows the association between household income and inadequate nutrient intake as an average of 2 days with school lunches. Children from households with a high income had significantly lower rates of nutrient shortages only for vitamin D and Mn compared with the rates for households with a middle income (*P* = 0.026 and *P* = 0.030, respectively). The low-income group showed a significantly higher rate of nutrient shortage for pantothenic acid compared with the middle-income group (*P* = 0.020), whereas the rates of shortages for other nutrients did not significantly differ between these groups.

**Table 3:** Logistic regression analysis of household income level and nutrient adequacy on 2 days with a school lunch: 5th grade children (10–11 years old) from 19 schools in four prefectures of Japan

	Cutoff value per day		Ref.	High (*n* = 323)			Middle (*n* = 194)	Low (*n* = 319)		
	Boys	Girls	type	OR	95% CI	*P*		OR	95% CI	*P*
Energy	<2,250 kcal	<2,100 kcal	EER	0.75	(0.50 – 1.12)	0.158	Ref	0.96	(0.63 – 1.47)	0.861
Protein	>20% E	>20% E	DG	NA	NA – NA	NA	Ref	NA	NA – NA	NA
	<13% E	<13% E	DG	0.74	(0.36 – 1.52)	0.417	Ref	0.70	(0.33 – 1.51)	0.367
	<40 g	<40 g	EAR	NA	NA – NA	NA	Ref	0.25	(0.01 – 7.23)	0.417
Fat	>30% E	>30% E	DG	0.90	(0.62 – 1.31)	0.591	Ref	0.74	(0.51 – 1.09)	0.130
	<20% E	<20% E	DG	0.79	(0.21 – 2.91)	0.724	Ref	0.82	(0.20 – 3.35)	0.781
Carbohydrate	>60% E	>60% E	DG	0.77	(0.24 – 2.49)	0.663	Ref	0.66	(0.18 – 2.41)	0.533
	<50% E	<50% E	DG	1.28	(0.68 – 2.42)	0.446	Ref	1.12	(0.56 – 2.21)	0.753
Fiber	<13 g	<13 g	AI	0.85	(0.58 – 1.26)	0.428	Ref	1.15	(0.77 – 1.72)	0.485
Vitamin A	<450 µg RAE	<400 µg RAE	EAR	0.92	(0.52 – 1.62)	0.768	Ref	1.12	(0.64 – 1.97)	0.700
Vitamin D	<4.5 µg	<4.5 µg	AI	0.65	(0.44 – 0.95)	0.026	Ref	0.81	(0.54 – 1.19)	0.281
Vitamin E	<5.5 mg	<5.5 mg	AI	0.94	(0.61 – 1.43)	0.763	Ref	1.13	(0.73 – 1.74)	0.593
Vitamin K	<120 µg	<120 µg	AI	1.14	(0.64 – 2.02)	0.665	Ref	1.46	(0.82 – 2.59)	0.201
Vitamin B1	<1.0 mg	<0.9 mg	EAR	0.83	(0.45 – 1.52)	0.548	Ref	0.79	(0.42 – 1.47)	0.453
Vitamin B2	<1.1 mg	<1.1 mg	EAR	1.25	(0.67 – 2.35)	0.480	Ref	1.53	(0.81 – 2.88)	0.192
Niacin	<11 mg NE	<10 mg NE	EAR	NA	NA – NA	NA	Ref	NA	NA – NA	NA
Vitamin B6	<1.0 mg	<1.0 mg	EAR	1.12	(0.59 – 2.14)	0.732	Ref	1.52	(0.81 – 2.85)	0.188
Vitamin B12	<1.5 µg	<1.5 µg	EAR	0.18	(0.01 – 2.39)	0.194	Ref	NA	NA – NA	NA
Folate	<150 µg	<150 µg	EAR	NA	NA – NA	NA	Ref	2.74	(0.24 – 31.69)	0.419
Pantothenic acid	<6 mg	<6 mg	AI	1.07	(0.63 – 1.83)	0.804	Ref	1.87	(1.10 – 3.18)	0.020
Vitamin C	<60 mg	<60 mg	EAR	1.01	(0.45 – 2.29)	0.974	Ref	0.81	(0.36 – 1.85)	0.623
Salt	>6.5 g	>7.0 g	DG	1.90	(0.61 – 5.91)	0.265	Ref	0.79	(0.30 – 2.07)	0.634
K	<1,900 mg	<1,800 mg	AI	0.79	(0.26 – 2.44)	0.682	Ref	1.68	(0.62 – 4.53)	0.305
	<2,200 mg	<2,000 mg	DG	1.06	(0.55 – 2.04)	0.866	Ref	1.73	(0.93 – 3.22)	0.083
Ca	<600 mg	<600 mg	EAR	0.87	(0.58 – 1.32)	0.518	Ref	1.18	(0.78 – 1.78)	0.437
Mg	<180 mg	<180 mg	EAR	0.71	(0.32 – 1.56)	0.392	Ref	0.70	(0.32 – 1.53)	0.373
P	<1,100 mg	<1,000 mg	AI	1.03	(0.66 – 1.61)	0.904	Ref	1.47	(0.95 – 2.30)	0.086
Fe	<7.0 mg	<7.0 mg	EAR	0.78	(0.54 – 1.14)	0.198	Ref	1.03	(0.70 – 1.51)	0.881
Zn	<6.0 mg	<6.0 mg	EAR	0.68	(0.14 – 3.24)	0.630	Ref	0.88	(0.19 – 4.08)	0.872
Cu	<0.5 mg	<0.5 mg	EAR	NA	NA – NA	NA	Ref	NA	NA – NA	NA
Mn	<3.0 mg	<3.0 mg	AI	0.59	(0.37 – 0.95)	0.030	Ref	0.78	(0.47 – 1.29)	0.332

AI, adequate intake; CI, confidence interval; DG, tentative dietary goal for preventing non-communicable diseases; EAR, estimated average requirement; EER, estimated energy requirement; %E, percentage of energy; NE, niacin equivalent; OR, odds ratio; RAE, retinol activity equivalents; Ref. type, reference type.

Adjusted for prefecture (A, B, C, and D), school (19 schools), sex (men and women), BMI (continuous), exercise frequency (>5 days/week, 3–4 days/week, 1–2 days/week, and 0 day/week), and food allergy status (yes and no) of the child, and parental education (less than high school, high school, college, university/graduate school, and don’t know).

Results from the analysis of associations between household income and inadequate nutrient intake on the 2 days without a school lunch are shown in Table 4. There were no significant differences in the rates of nutrient shortage between children from households with a high income and those from households with a middle income. In contrast, children from low-income households showed a significantly lower rate of excessive fat intake in addition to having low carbohydrate intake compared with those from middle-income households (*P* = 0.043 and *P* = 0.039, respectively). The intakes of several vitamins and minerals also showed significantly higher rates of shortages in the low-income group than in the middle-income group: vitamin B_6_ (*P* = 0.004), pantothenic acid (*P* = 0.001), K (*P* = 0.001 and *P* < 0.001 when the AI and DG reference values were used, respectively), Mg (*P* = 0.006), P (*P* = 0.037), Fe (*P* < 0.001), and Zn (*P* = 0.015).

**Table 4:** Logistic regression analysis of household income level and inadequate nutrient intake on 2 days without a school lunch: 5th grade children (10–11 years old) from 19 schools in four prefectures of Japan

	Cutoff value per day		Ref.	High (*n* = 323)			Middle (*n* = 194)	Low (*n* = 319)		
	Boys	Girls	type	OR	95% CI	*P*		OR	95% CI	*P*
Energy	<2,250 kcal	<2,100 kcal	EER	0.78	(0.50 – 1.23)	0.281	Ref	1.15	(0.71 – 1.87)	0.569
Protein	>20% E	>20% E	DG	6.03	(0.70 – 52.26)	0.103	Ref	0.79	(0.07 – 9.40)	0.854
	<13% E	<13% E	DG	0.81	(0.51 – 1.29)	0.384	Ref	1.06	(0.66 – 1.68)	0.815
	<40 g	<40 g	EAR	0.62	(0.21 – 1.83)	0.384	Ref	0.85	(0.31 – 2.38)	0.761
Fat	>30% E	>30% E	DG	0.88	(0.61 – 1.28)	0.510	Ref	0.68	(0.46 – 0.99)	0.043
	<20% E	<20% E	DG	0.51	(0.19 – 1.37)	0.182	Ref	0.84	(0.32 – 2.18)	0.717
Carbohydrate	>60% E	>60% E	DG	0.96	(0.60 – 1.55)	0.873	Ref	1.09	(0.49 – 2.43)	0.841
	<50% E	<50% E	DG	0.67	(0.28 – 1.59)	0.360	Ref	0.57	(0.33 – 0.97)	0.039
Fiber	<13 g	<13 g	AI	1.38	(0.92 – 2.07)	0.116	Ref	1.49	(0.98 – 2.27)	0.060
Vitamin A	<450 µg RAE	<400 µg RAE	EAR	1.22	(0.84 – 1.77)	0.303	Ref	1.41	(0.96 – 2.08)	0.083
Vitamin D	<4.5 µg	<4.5 µg	AI	0.83	(0.57 – 1.20)	0.326	Ref	1.16	(0.79 – 1.71)	0.446
Vitamin E	<5.5 mg	<5.5 mg	AI	0.80	(0.54 – 1.19)	0.268	Ref	1.45	(0.98 – 2.15)	0.063
Vitamin K	<120 µg	<120 µg	AI	1.05	(0.71 – 1.55)	0.808	Ref	1.35	(0.91 – 2.00)	0.139
Vitamin B1	<1.0 mg	<0.9 mg	EAR	0.85	(0.59 – 1.22)	0.378	Ref	0.89	(0.61 – 1.30)	0.541
Vitamin B2	<1.1 mg	<1.1 mg	EAR	0.96	(0.66 – 1.38)	0.817	Ref	1.29	(0.88 – 1.89)	0.185
Niacin	<11 mg NE	<10 mg NE	EAR	0.40	(0.06 – 2.73)	0.352	Ref	0.14	(0.01 – 1.66)	0.120
Vitamin B6	<1.0 mg	<1.0 mg	EAR	1.28	(0.87 – 1.89)	0.212	Ref	1.79	(1.20 – 2.65)	0.004
Vitamin B12	<1.5 µg	<1.5 µg	EAR	1.13	(0.53 – 2.39)	0.755	Ref	0.95	(0.44 – 2.07)	0.899
Folate	<150 µg	<150 µg	EAR	0.59	(0.34 – 1.03)	0.064	Ref	1.24	(0.74 – 2.08)	0.417
Pantothenic acid	<6 mg	<6 mg	AI	1.19	(0.82 – 1.73)	0.351	Ref	1.92	(1.29 – 2.83)	0.001
Vitamin C	<60 mg	<60 mg	EAR	0.95	(0.64 – 1.41)	0.790	Ref	1.34	(0.90 – 2.01)	0.149
Salt	>6.5 g	>7.0 g	DG	0.99	(0.59 – 1.65)	0.960	Ref	1.23	(0.71 – 2.13)	0.455
K	<1,900 mg	<1,800 mg	AI	1.06	(0.71 – 1.57)	0.792	Ref	1.96	(1.32 – 2.93)	0.001
	<2,200 mg	<2,000 mg	DG	1.15	(0.79 – 1.67)	0.458	Ref	2.00	(1.36 – 2.94)	<0.001
Ca	<600 mg	<600 mg	EAR	1.18	(0.74 – 1.89)	0.486	Ref	1.45	(0.88 – 2.39)	0.144
Mg	<180 mg	<180 mg	EAR	1.05	(0.71 – 1.57)	0.799	Ref	1.75	(1.17 – 2.62)	0.006
P	<1,100 mg	<1,000 mg	AI	0.93	(0.64 – 1.36)	0.714	Ref	1.52	(1.03 – 2.26)	0.037
Fe	<7.0 mg	<7.0 mg	EAR	1.17	(0.80 – 1.70)	0.421	Ref	2.13	(1.42 – 3.18)	<0.001
Zn	<6.0 mg	<6.0 mg	EAR	0.99	(0.56 – 1.73)	0.960	Ref	1.93	(1.14 – 3.29)	0.015
Cu	<0.5 mg	<0.5 mg	EAR	0.86	(0.16 – 4.66)	0.863	Ref	0.39	(0.07 – 2.25)	0.290
Mn	<3.0 mg	<3.0 mg	AI	0.66	(0.38 – 1.16)	0.153	Ref	0.90	(0.50 – 1.65)	0.740

AI, adequate intake; CI, confidence interval; DG, tentative dietary goal for preventing non-communicable diseases; EAR, estimated average requirement; EER, estimated energy requirement; %E, percentage of energy; NE, niacin equivalent; OR, odds ratio; RAE, retinol activity equivalents; Ref. type, reference type.

Adjusted for prefecture (A, B, C, and D), school (19 schools), sex (men and women), BMI (continuous), exercise frequency (>5 days/week, 3–4 days/week, 1–2 days/week, and 0 day/week), and food allergy status (yes and no) of the child, and parental education (less than high school, high school, college, university/graduate school, and don’t know).

## Discussion

In the present study, we determined the association between household income and nutrient adequacy based on DRIs in Japanese school children using 4-day dietary records including 2 days with and 2 days without a school lunch. To the best of our knowledge, this is the first study that showed differences in the prevalence of nutrient adequacy of Japanese schoolchildren according to their household income by considering DRIs. The association between household income and mean intake of food groups and nutrients for children has been previously examined (13). We also found that the difference in the fulfillment probability of nutrient adequacy due to household income decreased on days with a school lunch. The days without a school lunch were weekends and the days with a school lunch were weekdays. Lifestyle factors varied between the weekdays and weekends, and in particular, differences in dietary intake between the weekdays and weekends that were caused by the availability of a school lunch influenced the adequacy of nutrition intake.

On days without a school lunch, the prevalence of nutrient shortages was significantly higher compared with those on days with a school lunch for most macro- and micronutrients among all three levels of household income. Children from households with a high income had significantly lower rates of nutrient shortages for vitamin D and Mn compared with those from households with a middle income. Additionally, there was a significantly higher rate of shortage in pantothenic acid in the low-income group than in the middle-income group on days with school lunches. On the other hand, on days without a school lunch, children from low-income households had a significantly lower rate of excessive fat intake in addition to a low carbohydrate intake, but had higher rates of shortages in vitamin B_6_, pantothenic acid, K, Mg, P, Fe, and Zn compared with children from middle-income households.

Shortages of nutrient intakes could be an obstacle for physical and mental growth of children during school age. Moreover, dietary habits formed during school age tend to persist throughout life (4). Therefore, it is necessary to provide support for improving the nutrient intake of children from households with low income to reduce the risk of future increases in morbidity and mortality (2). These findings provide evidence for the prevalence of inadequate nutrient intake among Japanese schoolchildren from households with low income, and highlight that the high prevalence of shortages for most nutrients on days without a school lunch can be attributed to the availability of a school lunch.

One possible reason why the prevalence of a shortage in nutrient intake was significantly higher on days without a school lunch compared with on days with a school lunch is that school lunches have an important role in achieving adequate nutrient intakes for Japanese schoolchildren. In Japan, during the fiscal year 2018, the number of schoolchildren was 6,427,867, and elementary schools provided school lunches to 99.1% of all schoolchildren and offered lunch service on approximately 190 days per year (15). The Standards for the School Lunch Program established by the School Lunch Program Act (16) specifies that daily school lunches must contain at least a third of children’s nutritional requirements per day. Certain micronutrients including vitamin A (≥40%), vitamin B_1_ (≥40%), vitamin B_2_ (≥40%), Ca (≥50%), Fe (≥40%), and fiber (≥40%) have stringent requirements (17) that are intended to increase intake and avoid shortages of nutrients that result from insufficient intakes at breakfast and dinner. Therefore, when Japanese children attend school on weekdays, the school lunches guarantee a sufficient nutrient intake for lunch, and some micronutrients are also supplemented to cover the shortages from breakfast and dinner. Asakura et al. also recently reported that schoolchildren had significantly different intakes for ≥60% of nutrients on 2 schooldays as compared with 1 non-school day, although they did not consider children’s household income (27). Therefore, it is necessary to monitor children’s nutrient adequacies on days without a school lunch regardless of their household income.

When looking at salt intake, over 90% of schoolchildren exceeded the DG of DRI (boys: >6.5 g/day, girls: >7.0 g/day) (14) regardless of school lunch or household income. Excessive salt intake by the Japanese is a national issue (28), including salt intake in schoolchildren. In addition, our current study showed that salt intake was significantly higher on weekdays with a school lunch compared with weekends without a school lunch. According to a report on the formulation of the Standards for the School Lunch Program (29), the amount of salt expected to be consumed at a school lunch is −0.1 g/meal in 5th grade (ages 10–11 years) children, which was calculated by subtracting the amount of salt intake other than lunch (i.e. breakfast, dinner, and snack), estimated by dietary surveys from the DG of DRI. However, under the present dietary habits in Japan, it is difficult to season foods without using salt. Therefore, the amount of salt per meal used for school lunch for 5th grade children is set to be less than 1/3 of DG, that is, less than 2.5 g/meal. This standard value has been reduced from 3.0 to 2.5 g/meal in the past (30, 31) due to the revision of DRI from the viewpoint of promoting salt reduction. However, it is not desirable that the intake of school lunches, which are the key to food and nutrition education that conveys the ideal dietary intake for schoolchildren, can be a factor in increasing daily salt intake. It is important that salt content in a school lunch be below the current standard value.

Moreover, it should be particularly noted that on days without a school lunch, children from low-income households had higher rates of shortages for nutrients that are essential for physical and mental development, including vitamin B_6_, pantothenic acid, K, Mg, P, Fe, and Zn, as shown in Table 4. Several factors likely contributed to the higher rate of nutrient shortages in children from low-income households on days without a school lunch. Not only was there no improvement in the children’s nutrient adequacy from a school lunch on those days, the following factors have also been elucidated, mainly by studies in Western countries. Food cost affects the choice of food purchase. Previous studies have shown positive relationships between the food cost per calorie and the energy densities of fruits, vegetables, meat, fish, and eggs, whereas inverse relationships have been observed for fat and sugar (32, 33). Previous studies of Japanese adults have also reported that low-SES individuals consume a lower-cost diet (34). Additionally, the amount of knowledge about food, nutrition, health, and their interrelationships was less in the low-SES group, which is associated with a low educational level (35). These difficulties regarding food purchasing and having inadequate knowledge about food and nutrition among low-income households are likely to contribute to the inadequate nutrient intake of children. Moreover, school lunch is provided for about 190 days, which is 52% of the year (15), thus a high risk of inadequate nutrient intakes in children from low-income household remains for 48% of the year. In particular, Japanese elementary schools generally have long vacations such as during summer (40 days), winter (about 10 days), and spring (about 10 days) (36). There is no provision of school lunches during these periods; therefore, it is required to carefully monitor the risk of chronic inadequate nutrient intake during these long vacations. Improvements in socioeconomic disparities such as encouraging broader use of food access and provision in the community including food banks and community meals, implementing subsidies and allowances for child-rearing households and low-income households by the government, and giving guidance about how to easily cook healthy foods at a low cost are necessary to improve nutrient adequacy and long-term health outlook of children from households with low income.

The present study had several limitations. First, the final sample might not be representative of the population of East Japan prefectures or the entire Japanese population because the number of participants was not based on a sample size calculation, although researchers were selected from four prefectures in which they worked. Second, we could not compare our results with the lunch intake of children who did not have a school lunch program because the evidence on nutrient intake from school lunch programs in Japan is sparse. Further studies are needed to clarify the actual nutrient intake from school lunch programs in Japan, and how school lunch contributes to the children’s nutrient adequacy. Third, the rate of participants, who completed the study and were analyzed, dropped to 57.8%, although 85.1% of the children and their guardians in the participating schools initially agreed to participate in our study. However, we consulted with local governments and called for all 5th grade children in each school to participate in this study, so that the distribution of household income would be as close as possible to that in the prior Japanese national survey (11). Finally, the dietary records and photographs at home were practiced by children and their guardians. All children were taught how to record food and photograph their meals using the same manual by trained researchers to achieve the best consistency possible among the subjects.

## Conclusion

In conclusion, our current study found an association between household income and nutrient adequacy among Japanese primary school children using DRIs, and also found that children from households with low income tend to a high prevalence of nutrient shortages due to their poor dietary intake quality. On days without a school lunch, children from households with low income had higher rates of nutrient shortages for several vitamins and minerals that are essential for physical and mental growth at school age. However, the rates of nutrient shortage for these nutrients showed no significant differences according to household income on days with a school lunch. This evidence has important implications that school lunches are important for achieving adequate nutrient intakes in schoolchildren and reduce disparities of adequate nutrient intake by household income levels. Establishing effective nutritional policy such as providing school lunches during weekends and vacations or alternative approach to school lunches that enable substantial nutrient supply is needed to enable the achievement of adequate nutrient intake for children regardless of socioeconomic disparities.

## Conflict of interest and funding

The authors declare no conflict of interest. This research was supported by a Health and Labour Sciences Research Grant (Comprehensive Research on Life-Style-Related Diseases Including Cardiovascular Diseases and Diabetes Mellitus [N.M.; grant number H24-H26 Jyunkankitou-Seisyu-Ippan-006]) and a Health, Labour and Welfare Policy Research Grant (Comprehensive Research on Life-Style-Related Diseases Including Cardiovascular Diseases and Diabetes Mellitus [N.M.; grant number 19FA2001]) by the Ministry of Health Labour and Welfare, Japan. The founding sponsors had no role in the design of the study; in the collection, analyses, or interpretation of data; in the writing of the manuscript; or in the decision to publish the results.
